# Predicting intracerebral hemorrhage after endovascular therapy for anterior circulation strokes using CT-ASPECT, CTP-ASPECT and DWI-ASPECT: Protocol for a systematic review

**DOI:** 10.1371/journal.pone.0306295

**Published:** 2024-07-25

**Authors:** Vincent Brissette, Chirayu Bhatt, Brian Dewar, Risa Shorr, Jordi Pardo, Robert Fahed, Michel Shamy, Dar Dowlatshahi

**Affiliations:** 1 Department of Medicine (Neurology), Faculty of Medicine, Ottawa, Ontario, Canada; 2 Ottawa Hospital Research Institute, University of Ottawa, Ottawa, Ontario, Canada; 3 Department of Medicine, Faculty of Medicine, University of Ottawa, Ottawa, Ontario, Canada; UCSF: University of California San Francisco, UNITED STATES

## Abstract

**Introduction:**

Over the last decade, there have been significant advances in treatments for anterior ischemic stroke, most notably endovascular thrombectomy (EVT). Despite the success of EVT on overall outcomes, intracerebral hemorrhage (ICH) is an important post-procedure complication, often associated with mortality and disability. Hence, predicting the risk of ICH can inform EVT decision making. The ASPECT score is used globally to predict patients’ prognosis post-reperfusion therapy. Our objective is to perform a systematic review to collect and synthesize data on the association between ASPECT scores on CT, CTP and DWI-MRI (CT-ASPECT, CTP-ASPECT, and DWI-ASPECT) and the risk of symptomatic ICH after EVT for anterior circulation strokes.

**Methods and analysis:**

We will conduct a broad search of various electronic databases (MEDLINE, EMBASE, CINAHL, PsycINFO, Web of Science, and the Cochrane Database of Systematic Reviews) to identify studies published after January 1st, 2012 (commonly accepted as the beginning of the modern EVT era based on availability of stent-retrievers). Two independent reviewers will screen and include studies evaluating associations between symptomatic ICH after thrombectomy and baseline CT-ASPECT, CTP-ASPECT and DWI-ASPECT scores. Data will be extracted to quantify the risk of sICH after EVT based on the ASPECT scoring.

**Trial registration:**

**PROSPERO registration number:** CRD42023459860.

## Introduction and rationale

Ischemic stroke is a significant cause of morbidity and mortality worldwide. There are over 7.6 million new ischemic strokes each year, with 3.3 million deaths and 63 million Disability-Adjusted Life Years (DALYs) lost [1]. Treatments for ischemic stroke have significantly improved over the last decades with the availability of thrombolysis [2, 3] and, most recently, endovascular thrombectomy (EVT) [4–7]. Despite the success of EVT for anterior circulation strokes on outcomes, this interventional technique is not exempt from complications as patients can develop intracerebral hemorrhage (ICH) post-procedure [8, 9]. The incidence of symptomatic ICH after EVT varies from 3.6% to 9.3% in published Randomized Controlled Trials (RCTs), and from 1.9% to 15.8% in non-RCTs [10]. Symptomatic ICH after EVT is associated with worse mortality and disability; [8, 11] while data on incidence and prevalence is not complete, one study showed a lower rate of favourable neurological outcome in patients with sICH than those without (6.5 vs 43.3%), and a mortality rate of 54.8% in patients with sICH compared to 25.4% in those without [12]. Furthermore, although non-statistically significant, recent data suggest a signal for increased sICH amongst large infarcts undergoing EVT, and for patients undergoing EVT and receiving thrombolysis together compared to EVT alone [13, 14]. More research is required to predict the risk of ICH after EVT with or without thrombolysis, which could inform EVT decision-making.

The Alberta Stroke Program Early CT Score (ASPECT) is commonly used to interpret CT scans (CT-ASPECT) in acute stroke management, given its role in predicting patients’ prognosis post-reperfusion therapy [15]. The ASPECT score has been transposed onto other imaging modalities such as CT-Perfusion scan (CTP-ASPECT) and MRI scan (DWI-ASPECT), where it has also been proven to be a reliable predictor of clinical outcomes [16, 17]. Individual studies suggest the ASPECT score may help predict the risk of post-thrombectomy ICH, but there have been no attempts to formally evaluate the existing literature. Our study seeks to synthesize the literature and determine if the baseline ASPECT score calculated from different imaging modalities can reliably predict risk of post-thrombectomy ICH in anterior circulation strokes. The objective of our systematic review is to report the association between ASPECT scores on CT, CTP or DWI (CT-ASPECT, CTP-ASPECT, and DWI-ASPECT) and the risk of symptomatic ICH after EVT for anterior circulation strokes.

## Material and methods

This a priori protocol for a systematic review was developed according to the Preferred Reporting Items for Systematic Review and Meta-Analysis Protocols guideline (PRISMA-P) [18]. This study was registered in PROSPERO under registration ID CRD42023459860.

### Eligibility criteria

#### Study characteristics

Our inclusion criteria for this systematic review were defined in accordance with the following elements:

*Population*. We will include published randomized controlled trials (RCTs) and prospective observational studies of patients aged of 18 years or older treated with EVT for anterior circulation ischemic strokes. Eligible studies must include patients diagnosed with ischemic stroke of the anterior circulation by imaging, either with a computed tomography (CT) scan, a CT-Perfusion (CTP) scan, and/or a diffusion weighted imaging (DWI) on magnetic resonance imaging (MRI) scan, and report CT-ASPECT, CTP-ASPECT, or DWI-ASPECT scores. We will restrict to studies using third-generation devices (stent-retrievers and large-bore aspiration catheters) [19]. Studies will be included irrespective of EVT success and concurrent thrombolysis therapy. Studies focused solely on posterior circulation stroke will be excluded as well.

*Exposure and comparator*. The ASPECT score is a 10-point quantitative score assessing the presence of early ischemic changes, with 10 being no evidence of early ischemic changes [15]. Given the ASPECT score is an ordinal scale, we define our exposure and comparator as any ASPECT score presented in patients judged to be eligible for thrombectomy. We will assess all described associations in studies looking at the risk of ICH for each imaging modality based on ASPECT scores (CT-ASPECT, CTP-ASPECT, DWI-ASPECT). Given the categorical nature of the ASPECT scores, we hope to evaluate if studies support a cut-off ASPECT score that is associated with a higher risk of ICH on each imaging modality and compare if each imaging modality agrees on this existing cut-off.

*Outcome*. Our primary outcome will be to evaluate existing associations between symptomatic intracerebral hemorrhage (sICH) after thrombectomy based on included studies’ definition in relations with CT-ASPECT, CTP-ASPECT and DWI-ASPECT scores. For our secondary outcomes, if possible and available, we will define and present data according to the sICH definition as recommended by Kummer et al. when developing the Heidelberg classification [20]: new intracranial hemorrhage detected by brain imaging with any of (1) ≥4 points total NIHSS change at the time of diagnosis of sICH compared to before clinical worsening, (2) ≥2 points change in one NIHSS subcategory, (3) a need for hemicraniectomy/intubation/EVD placement/major surgical or medical intervention, or (4) the absence of an alternative explanation for clinical worsening. We will also assess the association of asymptomatic ICH (aICH) in relations with the same scoring system, if available.

Data will be collected, where possible, on risk factors potentially associated with a higher risk of bleeding, such as having received IV thrombolysis, the etiology of the stroke, having known comorbidities as presented in Table 1, blood pressure and glucose at admission, collateral vessels status prior to EVT, or having been on anticoagulation or antiplatelet therapy before or around the time of the procedure. Supplemental identifiable factors that could help in predicting the risk of bleeding in selected patients undergoing thrombectomy could facilitate decision-making for physicians treating anterior circulation ischemic stroke patients.

**Table 1:** Characteristics of included patients in included randomized controlled trials and observational studies.

**Study population data**	Age data
	Gender data
	Race/Ethnicity
	National Institute of Health Stroke Scale (NIHSS) score upon presentation to hospital
	Blood pressure at admission
	Glucose at admission
	Does the patient have information available on whether collateral vessels are supplying the ischemic area from the blocked vessel?
	What are the aetiologies of the ischemic strokes? (i.e., atherosclerosis, cardio-embolic, thrombus)
	What are important comorbidities of patients? Do they have hypertension, hypercholesterolemia, or diabetes?
	What was the time since last seen well of patients?
	Were patients treated with IV thrombolysis (i.e., alteplase)?
	Did patients receive antiplatelets in-hospital within the first 24h?
	Were patients on antiplatelets therapy prior to coming to the hospital?
	Were patients on anticoagulation at presentation to the hospital, and if yes, were they reversed prior to being treated with thrombectomy?
	What thrombectomy devices were used to remove the clot?
	If available, what was the mTICI scores of treated patients?
	Other relevant sociodemographics
**Outcome data**	Incidence of symptomatic ICH at 24h
	Type of symptomatic ICH using the Heidelberg Classification [20]
	Incidence of asymptomatic ICH at 24h
	Type of asymptomatic ICH using the Heidelberg Classification [20]
	Reported associations between ASPECT scores and ICH
	Existing ASPECT scores cut-off associated with a higher risk of ICH, if available
**Available imaging data**	Any imaging scoring linked to ICH in the published data

*Study selection*. Eligible studies included will be randomized controlled trials and prospective observational studies published after the publication of the SWIFT-PRIME trial [21] and the TREVO 2 trial [22], published on August 26^th^ and October 6^th^, 2012, respectively. Authors decided to use January 1^st^, 2012, as a cut-off in our screening given studies in preceding years did not use third-generation mechanical thrombectomy devices [4], which remain at the forefront of current intraarterial therapies. We will exclude case series, case reports, and review articles. There will be no specific length of follow-up for RCTs and observational studies to be included. However, at the data level, the length of follow-up will be 24h from the thrombectomy procedure for individual patients, given a repeat imaging is standardly performed to rule out hemorrhagic transformation of ischemic stroke at this time mark. Studies including patients with ICH happening only after 24h will be excluded.

#### Report characteristics

We will aim to include articles in languages other than English or French using automatic translation software. Abstracts without full text will be excluded, as will protocols and supplements. We will however consider protocols, supplement data and unsuccessfully translated materials to evaluate the risk of publications bias.

### Information sources and search strategy

We will execute our search strategy in MEDLINE, EMBASE, CINAHL, PsycINFO, Web of Science, and the Cochrane Database of Systematic Reviews. We will also search the collection of the University of Ottawa Health Sciences Library as well as any associated grey literature. Finally, the references of each included work will be searched for other potential materials to include.

### Search strategy

Full search strategy for all databases above are included in the S1 File. (Sig 1 Form 1) Our team has consulted with a librarian who created the search strategy. The following key terms using text and Mesh words were used to formulate our Search Strategy: (1) Stroke terms: ischemic stroke or infarction, anterior cerebral, anterior stroke, large vessel occlusion; (2) Endovascular terms: endovascular procedures or therapy, thrombectomy, mechanical thrombolysis; (3) Aspect: CT aspect, CTP aspect or DWI aspect, aspect score, Alberta stroke program early computer tomography score, computed tomography, computer tomography angiography, magnetic resonance imaging, diffusion magnetic resonance imaging, magnetic resonance angiography; (4) Prediction: predictive value of tests, observer variation, risk, prediction, predict.

### Study selection

We will use the Covidence Software Program to screen and manage selected records and data.

Two independent reviewers will perform the screening process, divided into two major steps. They will refer to the flowchart in Table 2 to ensure completeness and thoroughness. To maximize inter-rater reliability, reviewers will compare the first 50 articles screened, address any areas where screeners disagree on the interpretation of adjudication criteria and may clarify any misunderstandings. Inter-rater kappa between the two reviewers for the first 50 articles will be calculated. The screening criteria may be amended to document these clarifications.

1. For the initial step of the screening process, both reviewers will screen the article title and abstract to decide of its eligibility for inclusion. Articles included will be further assessed in the second step, others will be excluded.
2. For the second step of the screening process, both reviewers will independently screen the full text of each article. Articles judged eligible will be moved to the data extraction process.

Disagreements between reviewers in step 1 or 2 will first be resolved via consensus, and if needed, by a third independent, subsequently.

**Table 2:** Screening flowchart.

**1. Is the study’s language other than English or French?** a. If yes, stop and translate the article to English with automatic translation. Then, continue to the next step. b. If no, continue to the next step. **2. Is this study an abstract without full text, a protocol, supplement, or an article in press?** a. If yes, stop and exclude the article. b. If no, continue to the next step. **3. Is this study a randomized controlled trial or a prospective observational study?** a. If yes, continue to the next step. b. If no, stop and exclude the article. **4. Does the study include patients who have had ischemic strokes of the anterior circulation?** a. If yes, continue to the next step. b. If no, stop and exclude the article. **5. Was the diagnosis confirmed with CT scan, CTP scan, and/or DWI-MRI scan?** a. If yes, continue to the next step. b. If no, stop and exclude the article. **6. Were included patients treated with thrombectomy?** a. If yes, continue to the next step. b. If no, stop and exclude the article. **7. Were included patients followed for at least 24h?** a. If yes, continue to the next step. b. If no, stop and exclude the article. **8. Was the outcome of symptomatic ICH by ASPECT scores (CT-ASPECT, CTP-ASPECT and/or DWI-ASPECT) presented in studies?** a. If yes, article is eligible for inclusion. b. If no, stop and exclude the article.

### Data collection process

We will collect data both at the article level and at the data level. Two independent reviewers will perform the data collection independently. We created a data extraction form, (Sig 2 Form 2) that we will pilot for consistency and refine if required, using the Data Collection Form model available from Cochrane [23]. We will extract data using the Microsoft Excel software. Conflicts will be resolved by consensus, or if not possible, adjudicated by a third reviewer. When the required data is not available, we will contact the authors by e-mail of the included studies for additional information. If there is no response, we will send two reminders.

### Data items

First, we will collect basic characteristics of the articles as defined in Table 3. Simultaneously, we will extract the data from included studies. A priori, we will try to collect as much data as possible from the published articles and their available materials as defined in Table 1. Please refer to the data extraction form in the Supplemental Material section for more information. Secondly, in the event not enough data is available, we may ask authors of each included article to extract the missing information presented in Table 1, including raw data if necessary. However, we acknowledge that this may be difficult and not readily possible.

**Table 3:** Randomized controlled trials and observational studies basic characteristics.

**Bibliographic data**	Article name
	First author’s last name
	Publication Year
	Country
	Funding source
**Study design**	Length of trial
	Randomization process (applicable to RCTs solely)
	Unit of allocation (applicable to RCTs solely)
	Number of included centers
	Inclusion criteria
	Primary outcomes
	Secondary outcomes
	Safety variables
**Imaging modalities used to assess patients**	What was/were the imaging modality(ies) used to assess patients at their presentation to hospital?

We also acknowledge that some individual patient data may be missing or not documented and will account of missing data entries in our statistical analyses. There are no external sources of funding for this study.

### Risk of bias in individual studies

We acknowledge existing bias that can be present in randomized designs, such as reporting bias, attrition bias, and selection bias. Given that our review will focus on randomized controlled trials and observational studies, we will assess the risk of bias in individual studies using the Cochrane Risk-of-Bias Tool for Randomized Trials (RoB 2) [24] and the Risk of Bias in Non-Randomized Studies of Exposure (ROBINS-E) tool [25]. We will pilot the risk of bias assessment with the different tools in 2–3 studies to increase interrater agreement. Assessments will be done in duplicate, and disagreements will be resolved by consensus. A third author will adjudicate a decision if consensus cannot be reached. We will use the robvis tool to help visualize risk-of-bias assessments [26]. As previously mentioned, we will evaluate the risk of selective outcome reporting and publication bias by considering protocols, supplement data and unsuccessfully translated materials.

### Summary measures and synthesis of results

We will provide descriptive data on how many studies were screened, excluded, and included. We will provide descriptive demographic and clinical characteristics of the included studies. In the first part, we will present incidence rates of symptomatic ICH by ASPECT scores for each of the three imaging modalities (CT scan, CTP scan, DWI-MRI scan). We will initially provide descriptive information on incidence rates presented in included studies. If possible, we will try to meta-analyse the available data by ASPECT score to present an overall count and rate for each imaging modality using Poisson regressions. We will then present number and percentages of the risk of having an ICH post-thrombectomy based on presented and available ASPECT scores within each individual imaging category using χ2 test. We will interpret clinical and statistical significance using a p-value<0.05. We will use the same process to analyse the risk of our secondary outcome, asymptomatic ICH. Subgroup analysis will be performed when possible, using Odds ratio (ORs) and their 95% CI, and inter-agreement between imaging modalities using Kappa statistics will be assessed if possible. When sufficient data will be available, we will perform a subgroup analysis of the main outcome of symptomatic ICH by ASPECT score for each imaging modality. If sufficient data are available, subgroup analysis of the following will be performed using Odds ratio (ORs) and their 95% CI: male patients; female patients; age; study design (RCTs versus observational studies); patients with hypertension, diabetes, and hypercholesterolemia; patients on anticoagulation therapy; patients on antithrombotic therapy; risk of symptomatic and asymptomatic ICH by thrombectomy devices; patients who received IV thrombolysis; time since last seen well; and by stroke etiology. Similarly, incidence rates and risk measures will be calculated and presented, if available.

### Meta-bias(es)

If we include ten or more studies, we will assess the presence of publication bias using a funnel plot, as suggested by the Cochrane Handbook. If selective outcome reported is identified or suspected, we will assess its impact by modeling potential results. We will also quantitatively assess the presence of publication bias using Egger’s test. If possible, we will consider doing a meta-regression analysis using risk of bias as a potential predictor.

### Confidence in cumulative evidence

We will use GRADE guidelines [27] to assess the certainty of evidence of our summary estimate, and present our results in a summary of findings table.

## Supporting information

S1 Checklist PRISMA-P 2015 checklist. (DOCX)

S1 File Form 1. Search strategy. (DOCX)

S2 File Form 2. Data collection and extraction form. (DOCX)
