# Supplementary material for: Predicting intracerebral hemorrhage after endovascular therapy for anterior circulation strokes using CT-ASPECT, CTP-ASPECT and DWI-ASPECT: Protocol for a systematic review
Source: PLoS One. 2024 Jul 25;19(7):e0306295. doi: 10.1371/journal.pone.0306295 (PMC11271905; doi:10.1371/journal.pone.0306295)
Supplement: S1 File — (DOCX) [file pone.0306295.s002.docx]

**Form 1. Search Strategy**

**MEDLINE**

1 exp Ischemic Stroke/ 45077

2 Infarction, Anterior Cerebral Artery/ 5655

3 (isch?em* adj2 stroke*).tw,kf. 244221

4 (anterior adj5 stroke*).tw,kf. 7304

5 (cortical adj5 stroke*).tw,kf. 4849

6 large vessel occlusion*.tw,kf. 13336

7 or/1-6 265210

8 Endovascular Procedures/ 60438

9 (endovascular adj2 (therap* or treatment*)).tw,kf. or endovascular.ti. 111837

10 exp Thrombectomy/ 54020

11 thrombectom*.tw,kf. 50908

12 (mechanical* adj2 thrombo*).tw,kf. 4220

13 or/8-12 192778

14 7 and 13 34328

15 ((ct or ctp or dwi) adj10 aspect*).tw,kf,kw. 6526

16 (aspect* adj2 scor*).tw,kf. 3886

17 (Alberta Stroke Program Early Computed Tomography Score* or Alberta Stroke Program Early CT Score*).tw,kf,kw. 2523

18 aspect*.ti. 354153

19 tomography, x-ray computed/ or computed tomography angiography/ 592646

20 (computed tomograph* or ct perfusion).tw,kf. 842985

21 Magnetic Resonance Imaging/ or Diffusion Magnetic Resonance Imaging/ or Magnetic Resonance Angiography/ 1292804

22 (mri or magnetic resonance imag*).tw,kf. 1407065

23 (dwi or diffusion weigh* imag*).tw,kf. 59184

24 or/15-23 3325762

25 14 and 24 11083

26 "Predictive Value of Tests"/ 415283

27 observer variation/ 68643

28 predict*.tw,kf. or scor*.ti. or observ*.ti. 6262829

29 (observ* adj2 varia*).tw,kf. 99340

30 risk factors/ 2084565

31 risk*.tw,kf. 8166357

32 or/26-31 13694542

33 25 and 32 5393

34 33 use medall 1558

35 limit 34 to yr="2012 -Current" 1512

36 limit 35 to dt=20230830-20240109 74

**EMBASE**

37 cerebrovascular accident/ or exp ischemic stroke/ 493490

38 brain ischemia/ or exp anterior circulation ischemia/ 228083

39 (stroke* or cerebrovascular accident* or brain isch?em* or brain infarct*).tw. 980509

40 large vessel occlusion*.tw. 12994

41 or/37-40 1160571

42 (endovascular adj2 (therap* or treatment*)).tw. or endovascular.ti. 108657

43 exp thrombectomy/ 54020

44 thrombectom*.tw. 47800

45 (mechanical* adj2 thrombo*).tw. 3979

46 or/42-45 165905

47 41 and 46 49486

48 computer assisted tomography/ or computed tomographic angiography/ 996727

49 computed tomograph*.tw. 800913

50 diffusion weighted imaging/ or magnetic resonance angiography/ or *nuclear magnetic resonance imaging/ 326132

51 (dwi or diffusion weigh* imag*).tw. 55769

52 ((ct or ctp or dwi) adj2 aspect*).tw. 2761

53 (aspect* adj2 scor*).tw. 3875

54 Alberta Stroke Program Early CT Score*.tw. 2009

55 aspect*.ti. 354153

56 or/48-55 2159655

57 47 and 56 12889

58 predict*.tw. 5633562

59 diagnostic accuracy/ 314380

60 accurac*.tw. 1443908

61 (scor* or observ*).ti. 748565

62 (observ* adj2 varia*).tw. 98322

63 prediction/ 557553

64 diagnostic test accuracy study/ 240638

65 "receiver operating characteristic"/ 291835

66 or/58-65 7666153

67 57 and 66 4152

68 limit 67 to yr="2012 -Current" 4016

69 conference abstract.pt. 5024130

70 68 not 69 3134

71 70 use emczd 2034

72 limit 71 to yr="2012 -Current" 2034

73 limit 72 to dc=20230830-20240109 187

**Cochrane Database of Systematic Reviews**

74 exp Ischemic Stroke/ 45077

75 Infarction, Anterior Cerebral Artery/ 5655

76 (isch?em* adj2 stroke*).tw,kw. 241125

77 (anterior adj5 stroke*).tw,kw. 7274

78 (cortical adj5 stroke*).tw,kw. 4831

79 large vessel occlusion*.tw,kw. 13306

80 or/74-79 263121

81 Endovascular Procedures/ 60438

82 (endovascular adj2 (therap* or treatment*)).tw,kf. or endovascular.ti. 111837

83 exp Thrombectomy/ 54020

84 thrombectom*.tw,kw. 50272

85 (mechanical* adj2 thrombo*).tw,kw. 4741

86 or/81-85 192951

87 80 and 86 33729

88 ((ct or ctp or dwi) adj10 aspect*).tw,kf,kw. 6526

89 (aspect* adj2 scor*).tw,kw. 3879

90 (Alberta Stroke Program Early Computed Tomography Score* or Alberta Stroke Program Early CT Score*).tw,kf,kw. 2523

91 aspect*.ti. 354153

92 tomography, x-ray computed/ or computed tomography angiography/ 592646

93 (computed tomograph* or ct perfusion).tw,kw. 833398

94 Magnetic Resonance Imaging/ or Diffusion Magnetic Resonance Imaging/ or Magnetic Resonance Angiography/ 1292804

95 (mri or magnetic resonance imag*).tw,kw. 1392120

96 (dwi or diffusion weigh* imag*).tw,kw. 59670

97 or/88-96 3311122

98 87 and 97 10901

99 "Predictive Value of Tests"/ 415283

100 observer variation/ 68643

101 predict*.tw,kw. or scor*.ti. or observ*.ti. 6256499

102 (observ* adj2 varia*).tw,kw. 98611

103 risk factors/ 2084565

104 risk*.tw,kw. 8160959

105 or/99-104 13686105

106 98 and 105 5327

107 conference proceeding.pt. 230423

108 106 not 107 5256

109 108 use cctr 160

110 limit 109 to yr="2023 -Current" 20

**PsycINFO**

111 cerebrovascular accidents/ or cerebral hemorrhage/ or exp cerebral ischemia/ 593428

112 ((isch?em* or corticol or anterior) adj2 stroke*).tw. 239464

113 large vessel occlusion*.tw. 12994

114 Anterior Cerebral Artery infarct*.tw. 185

115 or/111-114 679765

116 endovascular.tw. 172166

117 thrombectom*.tw. 47800

118 (mechanical* adj2 thrombo*).tw. 3979

119 116 or 117 or 118 208188

120 115 and 119 41374

121 ((ct or ctp or dwi) adj10 aspect*).tw. 6484

122 (aspect* adj2 scor*).tw. 3875

123 (Alberta Stroke Program Early Computed Tomography Score* or Alberta Stroke Program Early CT Score*).tw. 2499

124 aspect.ti. 23084

125 exp magnetic resonance imaging/ 1883812

126 exp tomography/ 3643541

127 (computed tomograph* or ct perfusion).tw. 805890

128 (mri or magnetic resonance imag*).tw. 1343253

129 (dwi or diffusion weigh* imag*).tw. 55769

130 or/121-129 4153716

131 120 and 130 14257

132 exp clinical validity/ 505

133 predict*.mp. or scor*.ti. or observ*.ti. 6491638

134 (observ* adj2 varia*).tw. 98322

135 interrater reliability/ or test reliability/ 94726

136 risk.mp. 9217351

137 or/132-136 14205562

138 131 and 137 6660

139 138 use psyh 59

140 limit 139 to yr="2012 -Current" 55

141 36 or 73 or 110 or 140 336

142 remove duplicates from 141 281

**CINAHL**

| # | Query | Limiters/Expanders | Results |
| --- | --- | --- | --- |
| S1 | (MH "Ischemic Stroke+") | Expanders - Apply equivalent subjects Search modes - Boolean/Phrase | 1,355 |
| S2 | TI (ischem* N2 stroke*) OR AB (ischem* N2 stroke*) OR TI (ischaem* N2 stroke*) OR AB (ischaem* N2 stroke*) | Expanders - Apply equivalent subjects Search modes - Boolean/Phrase | 27,498 |
| S3 | TI (anterior N5 stroke*) OR AB (anterior N5 stroke*) | Expanders - Apply equivalent subjects Search modes - Boolean/Phrase | 902 |
| S4 | TI (cortical N5 stroke*) OR AB (cortical N5 stroke*) | Expanders - Apply equivalent subjects Search modes - Boolean/Phrase | 547 |
| S5 | TI large vessel occlusion* OR AB large vessel occlusion* | Expanders - Apply equivalent subjects Search modes - Boolean/Phrase | 1,556 |
| S6 | S1 OR S2 OR S3 OR S4 OR S5 | Expanders - Apply equivalent subjects Search modes - Boolean/Phrase | 29,028 |
| S7 | (MH "Endovascular Procedures") OR (MH "Thrombectomy") | Expanders - Apply equivalent subjects Search modes - Boolean/Phrase | 5,107 |
| S8 | TI endovascular OR AB endovascular | Expanders - Apply equivalent subjects Search modes - Boolean/Phrase | 15,090 |
| S9 | TI thrombectom* OR AB thrombectom* | Expanders - Apply equivalent subjects Search modes - Boolean/Phrase | 4,530 |
| S10 | TI (mechanical* N2 thrombo*) OR AB (mechanical* N2 thrombo*) | Expanders - Apply equivalent subjects Search modes - Boolean/Phrase | 563 |
| S11 | S7 OR S8 OR S9 OR S10 | Expanders - Apply equivalent subjects Search modes - Boolean/Phrase | 19,685 |
| S12 | S6 AND S11 | Expanders - Apply equivalent subjects Search modes - Boolean/Phrase | 3,695 |
| S13 | TI ( (ct or ctp or dwi) N10 aspect*) ) OR AB ( (ct or ctp or dwi) N10 aspect*) ) | Expanders - Apply equivalent subjects Search modes - Boolean/Phrase | 582 |
| S14 | TI (aspect* N2 scor*) OR AB (aspect* N2 scor*) | Expanders - Apply equivalent subjects Search modes - Boolean/Phrase | 544 |
| S15 | TI ( (Alberta Stroke Program Early Computed Tomography Score* or Alberta Stroke Program Early CT Score*) ) OR AB ( (Alberta Stroke Program Early Computed Tomography Score* or Alberta Stroke Program Early CT Score*) ) | Expanders - Apply equivalent subjects Search modes - Boolean/Phrase | 409 |
| S16 | TI aspect* | Expanders - Apply equivalent subjects Search modes - Boolean/Phrase | 13,373 |
| S17 | (MH "Tomography, X-Ray Computed") OR (MH "Computed Tomography Angiography") | Expanders - Apply equivalent subjects Search modes - Boolean/Phrase | 118,406 |
| S18 | TI ( (computed tomograph* or ct perfusion) ) OR AB ( (computed tomograph* or ct perfusion) ) | Expanders - Apply equivalent subjects Search modes - Boolean/Phrase | 80,886 |
| S19 | (MH "Magnetic Resonance Imaging+") OR (MH "Magnetic Resonance Angiography") | Expanders - Apply equivalent subjects Search modes - Boolean/Phrase | 147,166 |
| S20 | TI ( (mri or magnetic resonance imag*) ) OR AB ( (mri or magnetic resonance imag*) ) | Expanders - Apply equivalent subjects Search modes - Boolean/Phrase | 113,642 |
| S21 | ( (dwi or diffusion weigh* imag*) ) AND ( (dwi or diffusion weigh* imag*) ) | Expanders - Apply equivalent subjects Search modes - Boolean/Phrase | 6,809 |
| S22 | S13 OR S14 OR S15 OR S16 OR S17 OR S18 OR S19 OR S20 OR S21 | Expanders - Apply equivalent subjects Search modes - Boolean/Phrase | 326,786 |
| S23 | S12 AND S22 | Expanders - Apply equivalent subjects Search modes - Boolean/Phrase | 1,042 |
| S24 | (MH "Predictive Value of Tests") | Expanders - Apply equivalent subjects Search modes - Boolean/Phrase | 57,830 |
| S25 | TI predict* OR AB predict* | Expanders - Apply equivalent subjects Search modes - Boolean/Phrase | 468,444 |
| S26 | TI scor* OR TI observ* | Expanders - Apply equivalent subjects Search modes - Boolean/Phrase | 76,975 |
| S27 | AB (observ* N2 varia*) | Expanders - Apply equivalent subjects Search modes - Boolean/Phrase | 6,871 |
| S28 | (MH "Risk Factors") | Expanders - Apply equivalent subjects Search modes - Boolean/Phrase | 200,157 |
| S29 | TI risk* OR AB risk* | Expanders - Apply equivalent subjects Search modes - Boolean/Phrase | 931,491 |
| S30 | S24 OR S25 OR S26 OR S27 OR S28 OR S29 | Expanders - Apply equivalent subjects Search modes - Boolean/Phrase | 1,391,856 |
| S31 | S23 AND S30 | Expanders - Apply equivalent subjects Search modes - Boolean/Phrase | 532 |
| S32 | S23 AND S30 | Limiters - Published Date: 20120101-20231231 Expanders - Apply equivalent subjects Search modes - Boolean/Phrase | 507 |

**Web of Science**

# Web of Science Search Strategy (v0.1) - 2024-01-09

# Database: Web of Science Core Collection

# Entitlements:

- WOS.SCI: 1900 to 2024

- WOS.AHCI: 1975 to 2024

- WOS.ESCI: 2005 to 2024

- WOS.ISTP: 1990 to 2024

- WOS.SSCI: 1900 to 2024

- WOS.ISSHP: 1990 to 2024

# Searches:

1: TS=(ischem* Near/5 stroke*) OR TS=(ischaem* NEAR/5 stroke*) Results: 128707

2: (TS=(anterior Near/5 stroke*)) OR TS=(corticol Near/5 stroke*) Results: 2607

3: TS=(large vessel* Near/3 occlusion*) Results: 5291

4: #3 OR #2 OR #1 Results: 130181

5: (TS=(endovascular)) OR TS=(thrombectom*) Results: 95700

6: TS=(mechanical* Near/2 thrombo*) Results: 2300

7: #5 OR #6 Results: 96979

8: #4 AND #7 Results: 15351

9: TS=(aspect* NEAR/2 scor*) Results: 1928

10: (TS=("Alberta Stroke Program Early Computed Tomography Score*")) OR TS=("Alberta Stroke Program Early CT Score*") Results: 945

11: TS=((ct or ctp or dwi) NEAR/10 aspect*) Results: 2465

12: (TS=("computed tomograph*")) OR TS=("ct perfusion") Results: 429788

13: (TS=(mri )) OR TS=("magnetic resonance imag*") Results: 604895

14: (TS=(dwi)) OR TS=("diffusion weigh* imag*") Results: 23459

15: #9 OR #10 OR #11 OR #12 OR #13 OR #14 Results: 980434

16: #8 AND #15 Results: 3674

17: ((TS=(predict*)) OR TS=(risk*)) OR TS=((observ* NEAR/2 varia*)) Results: 8455604

18: #16 AND #17 Results: 1901

19: #16 AND #17 and Meeting Abstract (Exclude – Document Types) Results: 1890

20: PY=(2012-2024) Results: 36543093

21: #19 AND #20 Results: 1820
