# Supplementary material for: Predicting intracerebral hemorrhage after endovascular therapy for anterior circulation strokes using CT-ASPECT, CTP-ASPECT and DWI-ASPECT: Protocol for a systematic review
Source: PLoS One. 2024 Jul 25;19(7):e0306295. doi: 10.1371/journal.pone.0306295 (PMC11271905; doi:10.1371/journal.pone.0306295)
Supplement: S2 File — (DOCX) [file pone.0306295.s003.docx]

**Form 2. Data Collection and Extraction Form**

**Study name:**

| Article title or ID |  |
| --- | --- |
| Study ID *(surname of first author and year)* |  |
| Notes | |

# General Information:

| Date form completed *(dd/mm/yyyy)* |  |
| --- | --- |
| Name/ID of person extracting data |  |
| Reference citation |  |
| Study author contact details |  |
| Publication type  *(e.g. full report, abstract, letter)* |  |
| Notes: | |

# Study eligibility:

| **Study Characteristics** | **Eligibility criteria** | | **Eligibility criteria met?** | | | **Location in text or source** |
| --- | --- | --- | --- | --- | --- | --- |
|  |  | | Yes | No | Unclear |  |
| Type of study | Randomised Controlled Trial | |  |  |  |  |
|  | Prospective observational study | |  |  |  |  |
| Participants’ characteristics |  | |  |  |  |  |
| Thrombectomy devices used |  | |  |  |  |  |
| Symptomatic ICH incidence/rate and numbers |  | |  |  |  |  |
| INCLUDE | | EXCLUDE | | | | |
| Reason for exclusion |  | | | | | |
| Notes: | | | | | | |

**DO NOT PROCEED IF STUDY EXCLUDED FROM REVIEW**

## Methods:

|  | **Descriptions as stated in report/paper** | | **Location in text or source** |
| --- | --- | --- | --- |
| First author’s last name |  | |  |
| Country involved |  | |  |
| Aim of study/objective |  | |  |
| Study design |  | |  |
| For RCTs only: unit of allocation |  | |  |
| Randomization process (for RCTs only) |  | |  |
| Number of included centers |  | |  |
| Start date |  | |  |
| End date |  | |  |
| Publication year |  | |  |
| Study’s primary outcome |  | |  |
| Study’s secondary outcome(s) |  | |  |
| Safety variables |  | |  |
| Imaging modality used to assess patients |  | |  |
| Duration of participation/follow-up |  | |  |
| Ethical approval needed/ obtained for study | Yes No Unclear |  |  |
| Notes: | | | |

## Participants

|  | **Description** | | **Location in text or source** |
| --- | --- | --- | --- |
| Population description |  | |  |
| Setting |  | |  |
| Inclusion criteria |  | |  |
| Exclusion criteria |  | |  |
| Method of recruitment of participants |  | |  |
| Informed consent obtained | Yes No Unclear |  |  |
| Total no. randomised (for RCT) |  | |  |
| Clusters (for RCT) |  | |  |
| Age |  | |  |
| Sex |  | |  |
| Race/Ethnicity |  | |  |
| Co-morbidities (hypertension, hypercholesterolemia, diabetes) |  | |  |
| NIHSS score at presentation |  | |  |
| Blood pressure at admission |  | |  |
| Glucose at admission |  | |  |
| Collateral vessels prior to EVT |  | |  |
| Ischemic stroke aetiology |  | |  |
| Time since last seen well |  | |  |
| Treated with IV thrombolysis or not |  | |  |
| Treated with antiplatelets within first 24h |  | |  |
| Being on antiplatelets prior to presenting to hospital |  | |  |
| Being on anticoagulation prior to presenting to hospital |  | |  |
| Thrombectomy device used |  | |  |
| mTICI score post-thrombectomy |  | |  |
| Other relevant sociodemographics |  | |  |
| Notes: | | | |

## Outcomes

|  | **Description as stated in report/paper** | **Location in text or source** |
| --- | --- | --- |
| Incidence of symptomatic ICH at 24h |  |  |
| Type of symptomatic ICH using the Heidelberg Classification |  |  |
| Incidence of asymptomatic ICH at 24h |  |  |
| Type of asymptomatic ICH using the Heidelberg classification |  |  |
| Reported associations between ASPECT scores and ICH |  |  |
| Existing ASPECT scores cut-off associated with a higher risk of ICH, if available |  |  |
| Any imaging scoring linked to ICH in the published data |  |  |

## Other

| Study funding sources |  |  |
| --- | --- | --- |
| Possible conflicts of interest |  |  |
| Notes: | | |
